# Editorial on plants as alternative hosts for human and animal pathogens

**DOI:** 10.3389/fmicb.2015.00397

**Published:** 2015-04-30

**Authors:** Nicola J. Holden, Robert W. Jackson, Adam Schikora

**Affiliations:** ^1^Human and Animal Pathogen - Plant Interactions, The James Hutton InstituteDundee, UK; ^2^School of Biological Sciences, University of ReadingReading, UK; ^3^Institute of Phytopathology and Applied Zoology, Justus Liebig University of GiessenGiessen, Germany

**Keywords:** *Salmonella enterica*, *Escherichia coli*, *Arabidopsis thaliana*, fresh produce, PAMP-triggered immunity, effectors, mRNA extraction, microbiome

Fresh produce, in the form of raw or minimally processed fruits and vegetables, is recognized to be an important source of food-borne disease. Sporadic cases or outbreaks can arise from bacterial, viral, or parasitic contamination. Importantly, while the latter both require animal hosts for proliferation and cannot grow on or within crop plants, bacteria can do so and are able to use plants as secondary hosts. This type of interaction means that human pathogenic bacteria need to undergo adaptation to the plant host, which presents an environment quite distinct to animals or humans. Important differences lie in both physiochemical and biological properties, e.g., physiology, immunity, native microflora, physical barriers, mobility, and temperature. There is now good published evidence to describe different aspects of the interactions between plants and human pathogenic bacteria, and the subsequent effects on the outcome of colonization. However, in comparison to the interactions between the pathogens and their animal hosts, for which we know a great deal about both host and microbial factors, this area is relatively new with many important knowledge gaps. Research in this area is of most relevance to food safety, since the results can be and indeed, are already applied by public health agencies and food producers. It also reveals fascinating aspects of the bacterial life-cycle that were hitherto under appreciated.

One of the important features of plant host colonization is the adaptation of pathogens to the host defense response. Innate immunity is the first line of defense with both physical barriers and biological recognition of human pathogens, and the different layers of the response are reviewed in Melotto et al. (2014). An intriguing aspect is the finding that some human pathogens are able to subvert the plant defense, shown using *Salmonella enterica* and *Arabidopsis* as an example (Garcia and Hirt, 2014). Perception of human pathogens is based on recognition of PAMPs (e.g., flagellin) similar to those in phytopathogens and other plant-associated bacteria; perhaps unsurprising given that the mechanisms of recognition tend to be based on evolutionary conserved proteins. What was unexpected was the fact that some *Salmonella* serovars encode flagellin variants that are no longer recognized by the plant, presumably as a result of evolutionary selection (Garcia and Hirt, 2014).

There are good parallels in microbial suppression of the innate response of animal and plant hosts, for example via effectors secreted through the type 3 secretion (T3S) apparatus. Despite structural differences in the apparatus between human- and phyto-pathogens, the general function of host subversion is shared. Commonalities (and differences) in which pathways in animal and plant hosts are targeted by human pathogens are highlighted in Brunner and Fraiture (2014), showing where there is evolutionary conservation in the cellular hubs. Detailed examination of the function for one of the T3S effectors from *S. enterica*, SpvC, shows that it targets a signaling pathway that is shared in plant and animal hosts (Neumann et al., 2014). This effector is a phosphothreonine lyase able to de-phosphorylate activated MAP kinases, therefore attenuating the immune response in *Arabidopsis.*

Examination of mechanisms of colonization shows that although some aspects are broadly the same, there are important differences in the interaction between *S. enterica* and either plant or animal hosts (Wiedemann et al., 2015). To better understand the molecular basis of the interaction, it is necessary to determine the protein-protein interactions (PPIs) between the pathogen and the plant host. An important comparison of PPIs between *Salmonella* and *Arabidopsis* proteins to known interactions between bacterial and animal proteins was presented in Schleker et al. (2015). In order to characterize the interactions on a global scale, an approach was used that combines several new as well as previously described algorithms predicting new PPIs (Kshirsagar et al., 2015). However, while this approach yields candidates worthy of further investigation, it also serves to highlight the paucity of reported functional data. To further explore the molecular interactions, methods must be developed that are appropriate to the bacteria-plant system, for example for the analysis of bacterial gene expression. An improved method for the extraction of RNA from mixed samples was presented in Holmes et al. (2014), which allows the analysis of expression patterns in bacteria and the plant host at the same time.

Colonization of plant hosts by human pathogens rarely, if ever, occurs in isolation and successful colonization is dependent on the ability of the bacteria to compete with the native microflora. Microbiome approaches show that crops not obviously associated with human pathogens may harbor potential pathogens. As shown with an example of grapevines, crop plants can support the endophytic growth of human pathogen, in this case *Propionibacterium acnes*, which probably arose from human contamination in the first place (Yousaf et al., 2014). Application of bulky organic fertilizers, such as manure, to crops may increase the chances of transmission of bacteria into the food chain. However, the potential for transmission clearly shows some specificity, not least from differences between species and sub-species of bacteria and the host plants (Hofmann et al., 2014). A combination of axenic system with susceptible plants (e.g., spinach) revealed that 400 bacteria in one ml are sufficient to successfully colonize the crop plant.

This collection of articles helps to highlight the underpinning mechanisms, but also shows the complexity of interactions between human pathogenic bacteria and plant hosts. It is becoming clear that it is not possible to apply a broad set of rules to these interactions: there is a great deal of specificity that depends on multiple factors. This is particularly important in the consideration of how to address issues related to food safety, for practices applied at the pre-harvest stage or for prevention of transmission and contamination during post-harvest processing.

## Conflict of interest statement

The authors declare that the research was conducted in the absence of any commercial or financial relationships that could be construed as a potential conflict of interest.
